# Platinum–gold nanoraspberries as effective photosensitizer in anticancer photothermal therapy

**DOI:** 10.1186/s12951-019-0539-2

**Published:** 2019-10-15

**Authors:** J. Depciuch, M. Stec, B. Klebowski, J. Baran, M. Parlinska-Wojtan

**Affiliations:** 1 Institute of Nuclear Physics Polish Academy of Sciences, 31-342 Krakow, Poland; 2 Department of Clinical Immunology, Institute of Pediatrics, Jagiellonian University Medical College, 30-663 Krakow, Poland

**Keywords:** Platinum–gold nanoraspberries, Photosensitizers, Anticancer photothermal therapy

## Abstract

**Background:**

New nanophotosensitizers for photothermal cancer therapy (PTT) are still sought. In this paper we propose fancy shaped, non agglomerated core/shell PtAu NRs nanoraspberries (PtAu NRs) as potential nanophotosensitizers in PTT.

**Results:**

Light microscopy images of two colon cancer cell lines (SW480, SW620) showed, that the laser irradiation combined with PtAu NRs caused visible changes in the cell morphology. Fourier Transform InfraRed (FTIR) and Raman spectroscopies showed chemical changes in the DNA, phospholipids, lipids and protein structures caused by laser irradiation in the presence of PtAu NRs. The MTS assay showed ~ 25% mortality of cancer cells due to the addition of PtAu NRs to the cell culture, while for laser irradiation combined with nanoparticles, the mortality of cancer cells increased to 65% for the 650 nm laser and to 60% for the 808 nm laser. The calculated photothermal conversion efficiency reached 62% and 51% for the 650 nm and 808 nm lasers, respectively.

**Conclusions:**

PtAu NRs could be applied as effective light-absorbers in the PTT anticancer therapy.

## Background

Nowadays, dynamic growth of research in nanotechnology for biomedical applications is observed. The main reason, are unique properties of nanoparticles when compared with bulk materials [1]. The scale decrease significantly modifies structural, optical, mechanical and electrical properties of these materials. Moreover, nanoparticles, having sizes similar to biomolecules, can pass through the cell lipid bilayer, offering the opportunity to use NPs in biological systems [2]. Consequently, nanoparticles are used as imaging agents, drug delivery systems or radiosensitizers in proton, radiation and photothermal therapy (PTT) [3–5]. In PTT, nanoparticles are delivered to the tumor cells and subsequently exposed to light at the NPs resonant energy, causing synchronized oscillation of the NPs conduction-band electrons, what results in heat production [6]. Thus the temperature in the tumor increases causing cellular damage and subsequent tumor regression. However, to increase the efficiency of thermal irradiation, the NPs, which are used in PTT, must absorb electromagnetic waves with lengths in the range between 650 and 1064 nm [7]. One of the most studied nanoparticles used for enhancement of PTT are metallic nanoparticles, especially gold nanoparticles (Au NPs) or platinum nanoparticles (Pt NPs) [8]. Features like extensive surface-to-volume ratio, the possibilities for tailoring charge, hydrophilicity and functionalization by surface chemistry make Au NPs effective photosensitizers used in PTT [9]. The platinum ions from the Pt nanoparticles, in combination with Au NPs, could be used as anticancer therapeutics with an effect similar to cisplatin. Porcel et al. suggested that platinum nanoparticles could be used for the enhancement of radiosensitization [10–12]. This approach enables spatially-targeted chemical activation of prodrugs and has no precedent among the therapeutic strategies offered by metal nanostructures. Moreover, due to high extinction coefficients in the NIR region of Au NPs and Pt NPs, these nanostructures can be used in photothermal therapy [13]. Notwithstanding, we have to be careful with the use of gold nanoparticles in cancer therapies, because it is still unknown whether the toxic effects caused by nanoparticles are life-threatening. There are a lot of studies, which showed non-toxicity of NPs, as well as high number of publications showing a cytotoxic effect of nanoparticles on the cells. Pfaller et al. [14] investigated the immunotoxicity, cytotoxicity and also genotoxicity effects of Au NPs on human cells. They studied three concentration of Au NPs, starting from 5.5 * 10^10^ and reaching 5.5 * 10^12^ NPs/mL using several tests. The obtained results showed, that in all three concentrations, Au NPs were non-toxic. However, Miller et al. [15] showed, that Au NPs can accumulate in blood vessels and causing their damage. It is difficult to directly compare the results about toxicity effect of nanoparticles obtained by others authors, because they used different nanoparticles, synthesized by different methods and using different reagents, which also have influence on the toxicity of NPs [16]. Indeed, Schmid et al. [17] showed, that the physico-chemical properties of the NPs and their specific surface area are dominating the cytotoxic effect, while the added Au NPs concentration is less important.

In the present study, the novel approach, was the combination of Au NPs with Pt NPs for obtaining good photothermal properties. For this purpose, the fancy shaped core/shell PtAu NRs nanoraspberries (PtAu NRs) were synthesized and they photothermal efficiency was investigated using two 650 nm and 808 nm wavelengths. Moreover, complexes changes in the morphology, chemistry and viability of cells cultured with PtAu NRs and irradiated as well as non-irradiated by two wavelengths, were shown in this paper. The chemical changes were investigated by FTIR and Raman spectroscopy and this is the first time, when these two methods were used for the determination of changes in the chemical compositions of cells cultured with NPs and subjected to laser irradiation. Furthermore, in this work we showed the values of photothermal conversion efficiency for the nanoparticles composed of two elements.

## Materials and methods

### Synthesis of NRs

#### Pt NPs synthesis

10 mL of aqueous solution of ascorbic acid (0.1 M) was placed in a water bath with magnetic stirrer (400 rpm) and heated to 100 °C. Then, 2 mL of aqueous solution of H_2_PtCl_4_ (0.02 M) was added into the above solution, turning the color of the solution to brown after 10 min.

#### PtAu nanoraspberries synthesis

The PtAu nanoraspberries were prepared by mixing 3 mL of Pt NPs, 10 mL deionized water and 1 mL of solution of sodium citrate. The reaction mixture was put in a water bath with stirring (260 rpm) and heated to 93 °C. Then, 200 µL of HAuCl_4_ solution was added and kept at 93 °C for 20 min. The color of the solution changed into orange.

### TEM characterization

Scanning transmission electron microscopy (STEM) combined with the high-angle annular dark-field detector (HAADF) operating in conventional and high-resolution modes, was used to analyze the morphology of the synthesized nanoparticles. The crystallographic structure was assessed by selected area electron diffraction (SAED) patterns obtained in the TEM mode. The chemical composition of the synthesized nanoparticles was analyzed by energy dispersive X-ray spectroscopy (EDS). The observations were performed on an aberration-corrected FEI Titan electron microscope operating at 300 kV equipped with a FEG cathode. The EDS mappings were done using a FEI Talos TEM, operating at 200 kV, equipped with a FEG cathode and four in-column EDS detectors (Super EDS system). The particle size distribution was evaluated from the HRSTEM images taken from different areas of the TEM grids. For each sample, the diameter of 100 nanoparticles was measured.

### X-ray diffraction

The phase composition of the obtained nanoparticles was analyzed with X-ray diffraction (XRD). The XRD measurements were performed on a two-circle laboratory diffractometer Panalytical X’Pert Pro using a Cu anode with λ_Kα1_ = 1.5406 Å and λ_Kα2_ = 1.5444 Å working at 40 kV and 30 mA. The nanoparticles were deposited onto a zero background holder, which was placed on a sample spinner. The data were collected in the range from 30 to 90° (2θ) at room temperature. The patterns were analyzed using Rietveld refinement through the Fullprof software, while for peak fitting the Thompson–Cox–Hasting pseudo-Voigt profile function was used and the background was fitted by a 6-coefficient polynomial function [18, 19].

### Laser devices and PTT simulation protocols

The simulation of the PTT on colon cancer cells and colon cancer cells cultured with PtAu NRs were conducted by low-intensity LED lasers with 650 and 808 nm wavelengths. An adjustable power supply was connected to the setup to enable the control of the power output of the laser, which additionally was calibrated using a power meter. The intensity of the laser was set to 100 mW/cm^2^ and the irradiation time was chosen to be 5 min, based on preliminary experiments of cancer cell irradiation during different times. These results are presented in Additional file 1.

### Cell culture

Colon cancer cell lines (SW480, SW620) were obtained due to courtesy of Prof. Caroline Dive, Paterson Institute for Cancer Research, University of Manchester. These cell lines were cultured in DMEM with high glucose content (Corning, NY, USA) at 37 °C in humidified atmosphere with 5% CO_2_. All media were supplemented with 10% fetal bovine serum (FBS, Biowest, Nuaille, France) and gentamicin (50 µg/mL), (PAN-Biotech, Aidenbach, Germany). The cells were cultured by bi-weekly passages and were regularly tested for *Mycoplasma* sp. contamination by PCR-ELISA kit (Roche, Mannheim, Germany) according to the manufacturers’ instruction.

### MTS assay

The cytotoxic activity of PtAu nanoraspberries against human colon cancer cells (SW480 and SW620) was determined by using 3-(4,5-dimethylthiazol-2-yl)-5-(3-carboxymethoxyphenyl)-2-(4-sulfophenyl)-2H-tetrazolium (MTS) assay (CellTiter 96^®^ AQueous One Solution Cell Proliferation Assay, Promega, Madison, WI). Briefly, the cells were cultured in flat-bottom 96-well plates (Sarstedt, Numbrecht, Germany) at a density of 1 × 10^4^/well in DMEM medium containing 10% FBS. After 24 h, 20 μL of PtAu NRs solution was added to the cells. After additional 24 h of culture, 20 µL of MTS (CellTiter 96^®^ Aqueous One Solution Cell Proliferation Assay, Promega) dye solution was added per well and incubated for 2 h. The quantity of formazan product, directly proportional to the number of living cells in culture, was detected by absorbance measurement at 490 nm with a 96-well plate reader (Spark^®^ Tecan, Mannedorf, Switzerland). SW480 and SW620 cancer cell lines cultured without PtAu NRs and without irradiation were used as control samples.

In the present study the following samples were investigated (Table 1).

**Table 1:** Investigated samples

Control	SW480	SW620
Investigated samples	SW480 + PtAu NRs	SW620 + PtAu NRs
	SW480 + laser 650 nm and 808 nm	SW620 + laser 650 nm and 808 nm
	SW480 + PtAu NRs + laser 650 nm and 808 nm	SW620 + PtAu NRs + laser 650 nm and 808 nm

### Light microscopy images of cells

The images of cells at 100× magnification were taken using an optical microscope Olympus IX70 (Olympus Corporation, Tokyo, Japan).

### Cell preparation for FTIR and FT-Raman measurements

For FTIR and FT-Raman spectra acquisition, the cells in 10^8^/mL concentration were centrifuged for 5 min at 3000 rpm. Subsequently, the cells were washed three times in isotonic solution (NaCl, 0.9%) to ensure a complete removal of trypsin and culture medium.

### FTIR spectroscopy

The measurements were carried out on an EXCALIBUR FTS-3000 spectrometer at room temperature. All spectra were recorded by attenuated total reflection (ATR) with a ZnSn crystal. 0.5 mL of cell containing solution was deposited on the ATR ZnSn crystal. FTIR spectra were recorded between 4000 and 500 cm^−1^. Each spectrum was obtained by averaging 64 scans recorded at a resolution of 4 cm^−1^. Baseline correction and normalization of the obtained spectra were performed using the OPUS software.

### FT-Raman spectroscopy

FT-Raman spectra were recorded using a Nicolet NXR 9650 FT-Raman Spectrometer equipped with an Nd:YAG laser (1064 nm) and a germanium detector. The measurements were performed in the range from 150 to 3.700 cm^−1^ with a laser power of 1 W. Unfocused laser beam was used with a diameter of approximately 100 μm and a spectral resolution of 8 cm^−1^. The Raman spectra were processed by the Omnic/Thermo Scientific software based on 128 scans.

### Statistical analysis

The obtained MTS assay results are represented as the mean ± SEM (the standard error of the mean). The quantitative results were finally compared with the T test. p value < 0.05 was considered to be statistically significant. Moreover, to obtain the information about the spectra variation depending on the type of measured samples, PCA was performed. The PCA procedure was done based on the selected spectral regions between 800 and 1800 cm^−1^ using the Past 3.0 software.

### Photothermal conversion efficiency of PtAu nanoraspberries

For photothermal conversion measurements, 0.3 mL of PtAu nanoraspberries containing solution were introduced into glass cuvettes. The solutions were laser irradiated at 650 nm and 808 nm wavelengths, in order to study the wavelength dependent photothermal conversion. The temperature evolution was recorded by a digital multimeter connected to a small Pt-100 thermo-resistor located inside the cuvette. The PtAu NRs were irradiated for 5 min. To apply the time constant method, we took into account that the time evolution of the temperature after the laser was switched off, can be described by the following equation:

1 $$\eta = \frac{{\left( {c_{w} m_{w} + c_{nPt} m_{nPt} + c_{nAu} m_{nAu} } \right)\Delta T}}{IA\Delta t}*100\%$$

where *c*_*w*_, *c*_*nPt*_ and *c*_*nAu*_ are the specific heats of water, Pt and Au, respectively; *mw*, *mnPt* and *mnAu* are the masses of water, Pt and Au, respectively; *ΔT* is the temperature rise in the *Δt* time interval, *A* is the illumination area of the fluid in the experiment and *I* is the laser incident power. Moreover, to obtain the information about the photothermal efficiency of the nanoparticles, the following equation was used:

2 $$\eta_{\text{NRs}} = \frac{{\left( {c_{w} m_{w} + c_{nPt} m_{nPt} + c_{nAu} m_{nAu} } \right)\Delta T_{1} - c_{w} m_{w } \Delta T_{2} }}{{m_{nPt} + m_{nAu} \Delta t}}*100\%$$

where *ΔT*_*1*_ is the difference of the PtAu NRs solution temperature before and after laser irradiation, *ΔT*_*2*_ is a difference of the water solution temperature before and after laser irradiation.

## Results

### Morphology and structure of PtAu NRs

**Fig. 1:**
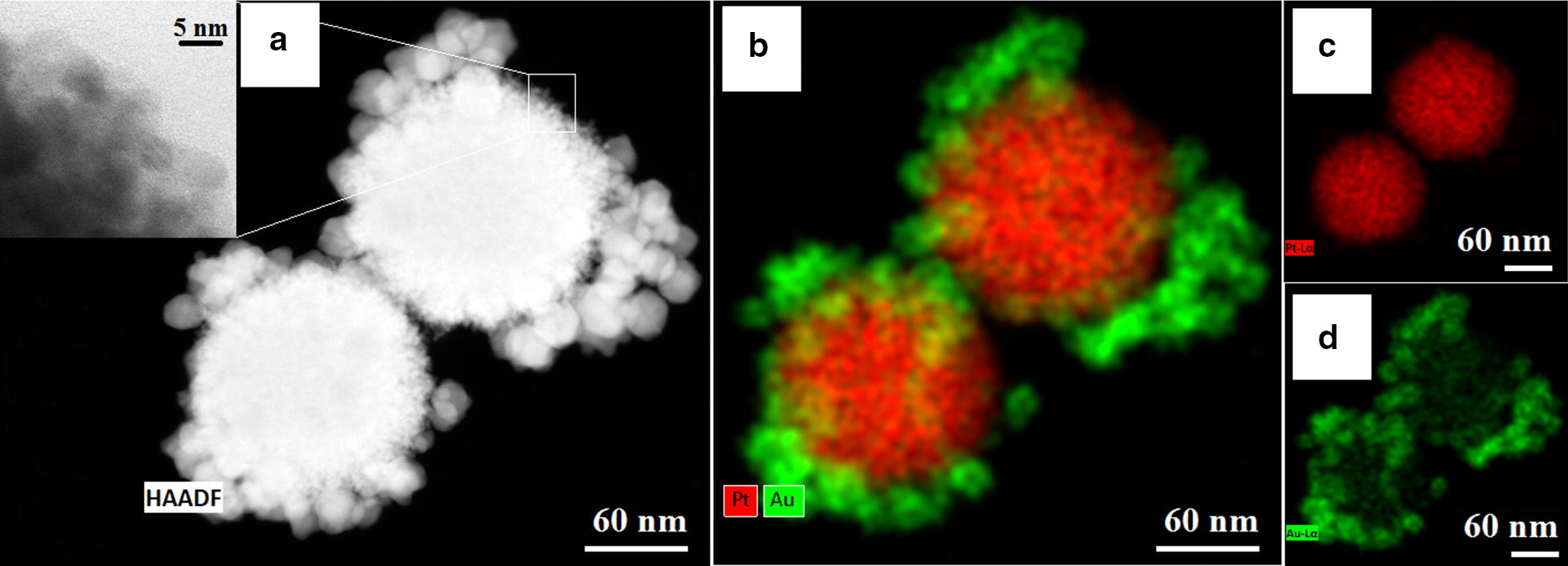
STEM image of PtAu NRs (**a**); EDS distribution map of Pt and Au in the NRs (**b**); individual EDS distribution maps: Pt—green (**c**) and Au—red (**d**) distribution EDS maps

The morphology and the chemistry of PtAu NRs were studied by electron microscopy. In Fig. 1, a STEM HAADF image of two nanoparticles (Fig. 1a) with the corresponding EDX maps (Fig. 1b) of Pt (c) and Au (d) distribution are presented. The Pt and Au NPs can be distinguished due to the Z-contrast differences of the nanoparticles, which are arranged similarly to core/shell raspberry structures, with a dozen of gold nanoparticles surrounding individual Pt nanoparticles. The shapes of both Au and Pt NPs are spherical, however the surface of the gold NPs is smooth, while the surface of the Pt NPs is furry-like. A larger magnification STEM image of the Pt nanoparticle, Fig. 1a, reveals that it consists of < 5 nm small Pt NPs, which agglomerated into a spherical object. Furthermore, the size of Au NPs is around 10 nm, while the size of the agglomerated Pt NPs is eight times larger. It is very important that Au NPs, core Pt NPs and also PtAu NRs had a uniform size distribution and did not agglomerate.

**Fig. 2:**
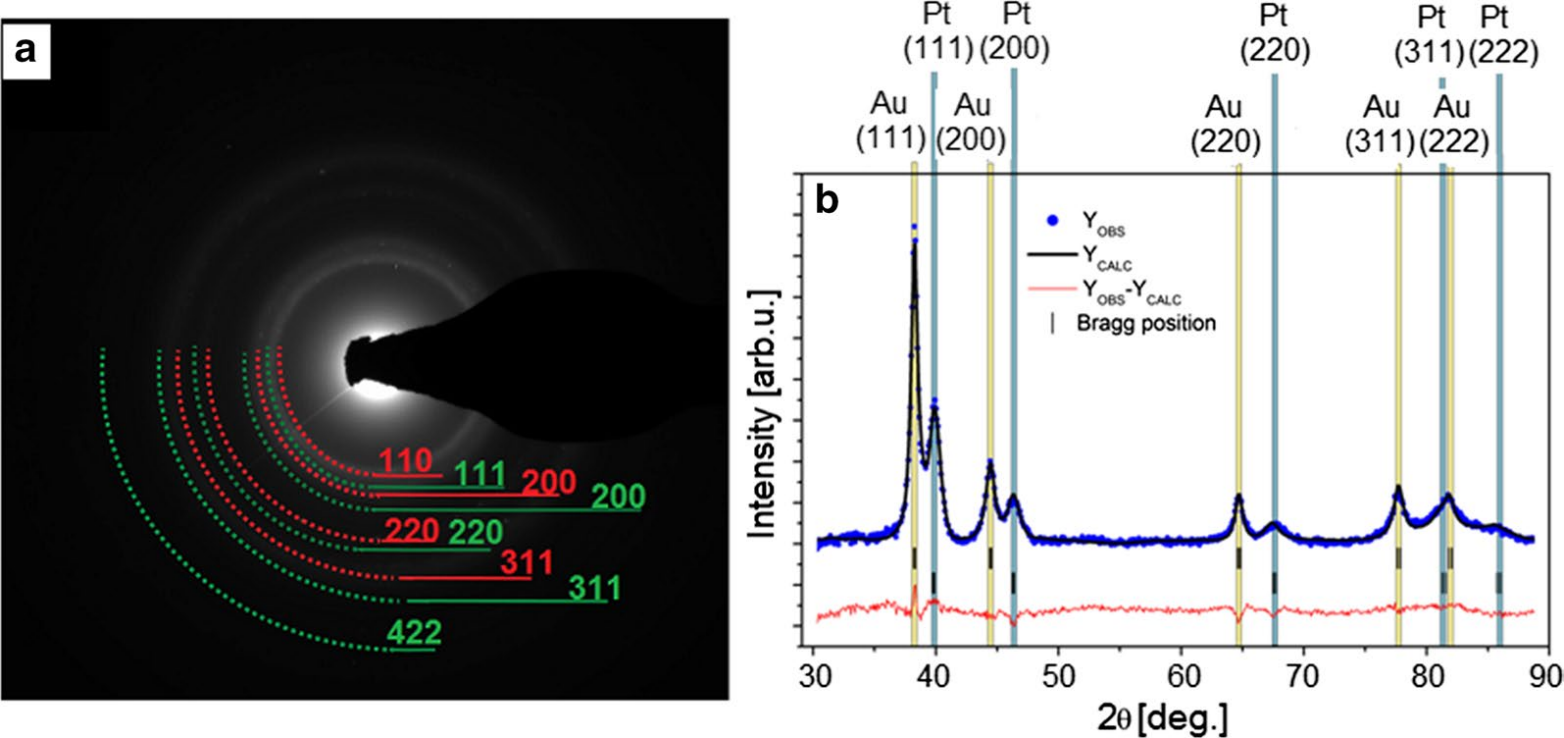
SAED patterns of the PtAu NRs indexed with lattice parameters of Pt (red circles) and Au (green circles) (**a**); XRD recorded for PtAu NRs (**b**). The XRD pattern was refined with Rietveld method, where dots represent the experimental data, black line—the calculated plot, red line—the difference between the observed and the calculated data. Tick marks show the positions of the allowed Bragg reflections

The structural information concerning the PtAu NRs was obtained from TEM selected area electron diffraction (SAED). The SAED pattern (Fig. 2a) confirms that the NRs have a crystalline structure. The rings on SAED (Fig. 2a) patterns could be attributed to the (111), (200), (220), (311) and (222) lattice planes of Au and Pt nanocrystals with face-centered cubic lattice [20]. Due to the large size of the Pt NPs (> 140 nm), the SAED pattern should contain some spots, however it is formed of well-defined, continuous rings. This is related to the fact that the Pt NPs consist of an aggregation of small, < 5 nm, NPs, which scatter the incident electron beam uniformly forming regular diffraction rings. These structural findings were confirmed by analysis of XRD standard Bragg reflections (Fig. 2b), which could be assigned to fcc gold and fcc platinum [20–22]. The calculated lattice constants (a_Au_ = 4.076 Å and a_Pt_ = 3.922 Å with uncertainties ~ 0.001 Å) were in a good agreement with lattice constants of Au and Pt nanoparticles known from the literature [23, 24]. In the present study, the fabricated nanoparticles consisted of two crystal phases (gold and platinum) and no alloy formation was identified by XRD. From TEM images it was found that Pt nanoparticles were a few times larger than the surrounding Au nanoparticles. Remembering that the Pt cores are composed of tiny nanoparticles, which aggregate into larger spherical objects, it is obvious that the full width at half-maximum (FWHM) of the peaks from the Pt phase is larger than the FWHM from Au shell. Thus the XRD patterns nicely confirm that the size of crystallites in platinum core was smaller than size of the Au NPs [25]. The average coherent scattering lengths (〈D〉) for gold and platinum phases, which we assumed to be equal to crystallite sizes, were estimated in the framework of micro-structural analysis from Fullprof and were around 〈D_Au_〉 ≈ 10 nm and 〈D_Pt_〉 ≈ 5 nm. In conclusion, TEM and XRD analysis evidenced that the fabricated PtAu nanoraspberries can be described as large agglomerates of small platinum crystallites forming the core, which are surrounded by well-crystallized gold nanoparticles forming a non-continuous shell.

### Morphological, chemical and viability changes in cancer cells induced only by the addition of PtAu NRs and by irradiation with 650 nm and 808 nm lasers in the presence of PtAu NRs

The obtained results showing morphological, chemical and viability changes in cancer cells caused by the addition of PtAu NRs, by irradiation with 650 nm and 808 nm lasers with and without PtAu NRs, were compared to control samples.

**Fig. 3:**
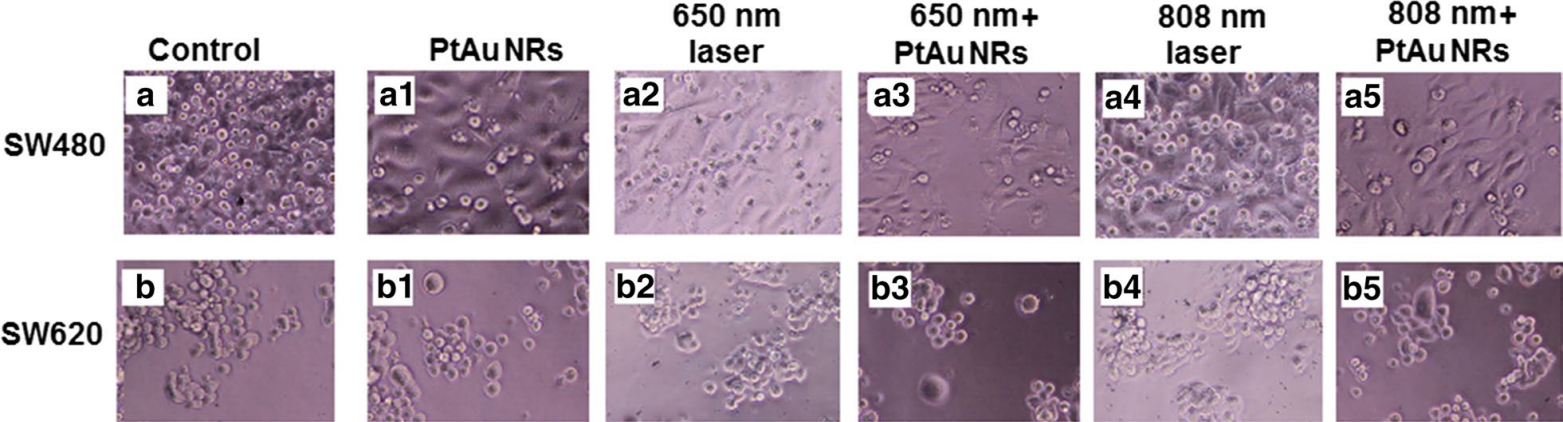
Light microscopy images of colon cancer cells morphology: control SW480 (**a**) and SW620 (**b**) cell lines; SW480 cells cultured with PtAu NRs (**a1**); SW480 cells cultured with PtAu NRs (**b1**); SW480 cells after 650 nm laser irradiation (**a2**) and SW620 cell line after 650 nm laser irradiation (**b2**); SW480 cells after 650 nm laser irradiation (**a3**) and SW620 cell line after 808 nm laser irradiation (**b3**); SW480 cells cultured with PtAu NRs and irradiated by the 808 nm laser (**a4**); SW620 cells cultured with PtAu NRs and irradiated by the 650 nm laser (**b4**); SW480 cells cultured with PtAu NRs and irradiated by the 808 nm laser (**a5**); SW620 cells cultured with PtAu NRs and irradiated by the 808 nm laser (**b5**)

The microscopy images of SW480 and SW620 cell lines cultures, showed that PtAu NRs addition (Fig. 3a1, b1), as well as laser irradiation in nanoraspberries presence (Fig. 3a3, b3, a5, b5), caused morphological changes in the cells, when comparing the images with those of control samples, Fig. 3a, b, respectively. Moreover, it was noticed, that PtAu NRs combined with laser irradiation caused more damage to the cells than PtAu NRs addition, because in the latter case, a larger number of cells survived, which was observed as cells adhesion to the base. Conversely, irradiation of cells cultured without PtAu NRs by lasers caused no visible changes in the morphology of these cells (Fig. 3a2, b2, a4, b4), in comparison with the control. This observation confirms, that the irradiation time of 5 min was chosen adequately, as it was intended not to destroy the cancer cells. Moreover, light microscopy images of cells showed, that generally higher mortality of the investigated SW480 samples was observed, in comparison with investigated SW620 cell lines samples.

**Fig. 4:**
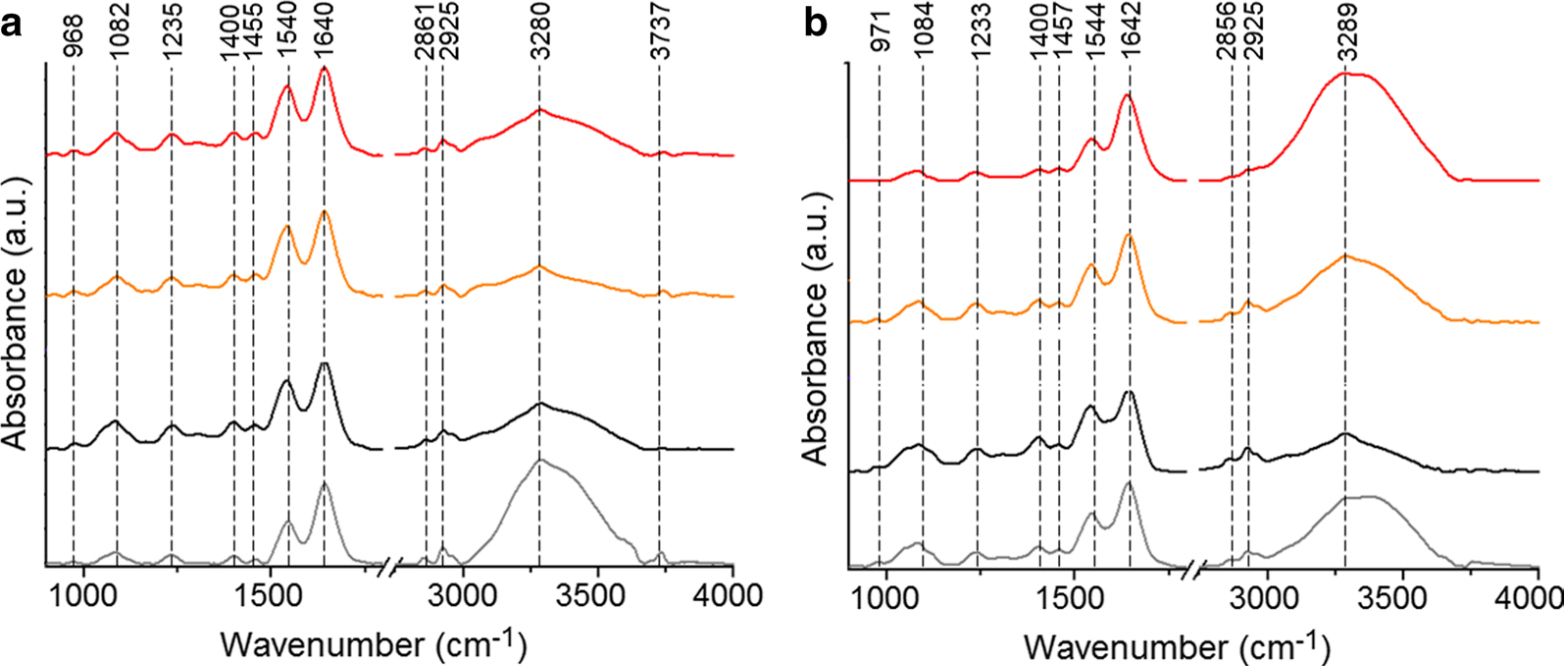
FTIR spectra of SW480 (**a**), SW620 (**b**) colon cancer cell lines: control (grey spectrum); cells cultured with the PtAu NRs (black spectrum); cells cultured with the PtAu NRs and irradiated by the 650 nm laser (orange spectrum); cells cultured with the PtAu NRs and irradiated by the 808 nm laser (red spectrum)

**Fig. 5:**
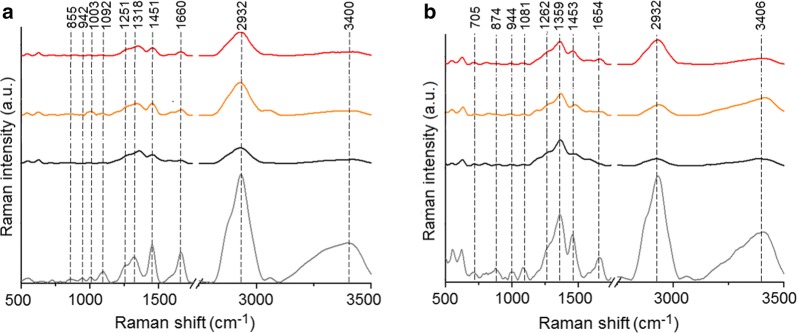
FT-Raman spectra of SW480 (**a**), SW620 (**b**) colon cancer cells line: control (grey spectrum); cells cultured with the PtAu NRs (black spectrum); cells cultured with the PtAu NRs and irradiated by the 650 nm laser (orange spectrum); cells cultured with the PtAu NRs and irradiated by the 808 nm laser (red spectrum)

For the first time we showed the chemical changes in the cells occurring after the addition of PtAu NRs, as well as after laser irradiation in the presence of nanoparticles, which were investigated by FTIR (Fig. 4) and FT-Raman (Fig. 5) spectroscopies. When we compare the FTIR (Fig. 4) and Raman (Fig. 5) spectra of cells cultured with PtAu NRs and irradiated by 650 nm and 808 nm lasers with spectra of control cells (grey spectra), differences in the values of maximum absorbances, as well as in the peak positions, were observed. Furthermore, in the Raman spectra absences of peaks were noticed in the cells cultured with nanoraspberries. The identified peaks for all the obtained FTIR and Raman spectra are presented in detail in Table 2.

**Table 2:** FTIR and Raman peaks positions in the analyzed samples with description of vibrations corresponding to the respective functional groups [26–41]

SW480	SW480 + PtAu NRs	SW480 + PtAu NRs + 650 nm	SW480 + PtAu NRs + 808 nm	SW620	SW620 + PtAu NRs	SW620 + PtAu NRs + 650 nm	SW620 + PtAu NRs + 808 nm	Vibrations
*FTIR spectroscopy peaks (cm* ^*−1*^ *)*								
968	971	974	974	977	974	971	970	PO_3_^−2^ group from DNA, RNA and phospholipids
1082	1084	1087	1086	1083	1082	1084	1081	C–O group from glycogen
1235	1236	1234	1235	1237	1236	1237	1235	Amide III
1400	1402	1400	1400	1402	1402	1401	1403	CH_2_ group from protein and lipids
1455	1458	1458	1458	1458	1455	1456	1457	CH_2_ group from cholesterol
1540	1545	1545	1546	1544	1534	1533	1534	Amide II
1640	1641	1642	1642	1642	1641	1642	1638	Amide I
2861	2860	2857	2858	2860	2862	2862	2864	Symmetric stretching vibrations of CH_2_
2925	2928	2925	2926	2927	2927	2927	2928	Asymmetric stretching vibrations of CH_2_
3280	3284	3289	3284	3296	3290	3288	3281	CH_3_ asymmetric stretching
3737	3738	3739	3741	3756	3727	3726	3738	Amide A and OH group from water
*Raman spectroscopy peaks (cm* ^*−1*^ *)*								
711	706	715	–	715	713	724	706	C–H in-plane bending mode
855	842	866	846	874	892	862	883	C–H in-plane bending mode
942	950	947	943	943	941	981	983	C–H in-plane bending mode of phenylalanine
1092	1078	1082	1078	1081	1074	1081	1075	Glucose, Triglycerides, C–C (lipid)
1251	1283	1282	1285	1286	1278	1274	1275	Amide III
1318	1341	1335	1350	1359	1362	1365	1359	CH_3_/CH_2_ twisting or bending mode of lipid/collagen
1451	1452	1451	1453	1453	1473	1467	1462	Fatty acids, CH_2_ (lipids and proteins)
1660	1661	1663	1661	1654	1691	1671	1663	Amide I
2932	2929	2928	2930	2932	2927	2937	2929	CH band of lipids
3400	3414	3420	3421	3417	3411	3416	3409	CH from cholesterol and cholesterol ester

All the obtained results for the SW480 cells were compared with control samples consisting of SW480, while the results obtained for the SW620 cells were compared with the SW620 cell line, respectively. In the FTIR spectra of both cell lines, the shift of the peak corresponding to the PO_3_^−2^ group from DNA, RNA and phospholipids, was visible. Moreover, the addition of PtAu NRs to the cells and subsequent laser irradiation caused changes in the amide I structure and in CH_3_ asymmetric stretching lipids vibrations in the SW480 and SW620 cells. Moreover, in the SW480 cells cultured with PtAu NRs, a shift of the peak originating from the C–O group from glycogen was induced by irradiation. A shift of the amide A peak was caused by the nanoraspberries addition to the SW620 cells and subsequent laser irradiation. Moreover, in cells with PtAu NRs irradiated using the 808 nm laser structural changes in the amide I were induced.

In the Raman spectra of both cell lines cultured with PtAu NRs irradiated and non-irradiated by 650 nm and 808 nm lasers, shift of peaks corresponding to the C–H in-plane bending mode and the amide III were observed. Moreover, in the Raman spectra of the SW480 and SW620 cells cultured with PtAu NRs and irradiated as well as non-irradiated by lasers, structural changes in glucose, triglycerides, collagen and cholesterol, were noticed. In the case of the SW620 cells cultured with PtAu NRs and laser irradiated, shift of peaks corresponding to CH_2_ fatty acid functional groups and amide I, were visible. Moreover, laser irradiation with PtAu NRs caused structural changes in SW620 cells in phenylalanine.

**Fig. 6:**
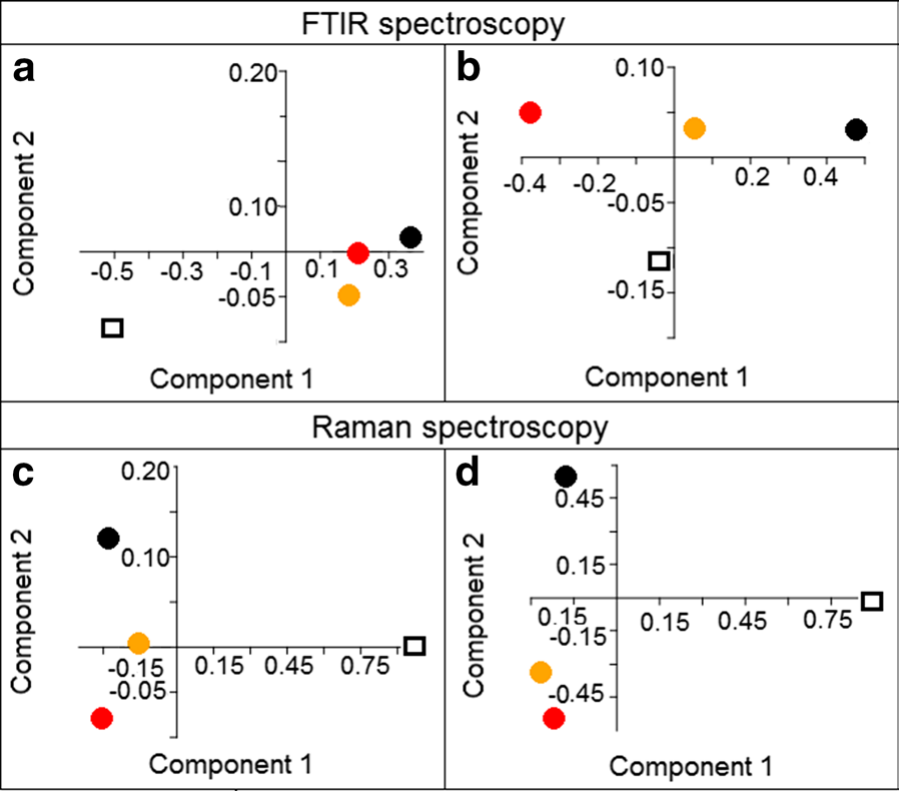
PCA analysis of FTIR and Raman spectra of: SW480 (**a** and **c**, respectively) and SW620 (**b** and **d**, respectively) cell lines. PCA was performed based on the selected spectral FTIR and Raman regions: between 800 and 1800 cm^−1^. The different colors of dots correspond to following samples: control (hollow squares); cells cultured with PtAu NRs (black dots); cells cultured with PtAu NRs and irradiated by the 650 nm light (orange dots); cells cultured with PtAu NRs and irradiated by the 808 nm wavelength (red dots)

When we compare the FTIR and FT-Raman spectra obtained for the investigated samples and the control samples, differences in the structure and values of absorbance, as well as in the Raman intensities, were observed. However, when we compare spectra obtained for cells cultured with PtAu NRs and subjected to laser irradiation, the FTIR and FT-Raman results are similar. Therefore, to obtain information about the significant differences between the spectra, PCA analysis was performed and the results are presented in Fig. 6.

PCA analysis of FTIR and Raman spectra of both cell lines (Fig. 6) showed, that the data obtained for the control samples is located in a different quarter than the data obtained for other samples. It means that the chemical changes caused by PtAu NRs and laser irradiation combined with nanoparticles are statistically significant. Moreover, PCA analysis of FTIR data for the SW480 cell line (Fig. 6a) and of Raman data for both cell lines (Fig. 6c, d) showed that the PtAu NRs caused other chemical changes, than these obtained for cells with nanoparticles irradiated by lasers. Furthermore, PCA analysis of these samples (Fig. 6a, c, d) showed that laser irradiation in the presence of PtAu NRs caused similar chemical changes in the cells, regardless which laser wavelength was used. Only for the FTIR data obtained from the SW620 cell line (Fig. 6b), PCA analysis showed that laser irradiation with PtAu NRs caused similar chemical changes to these obtained for cells cultured with nanoparticles. Moreover, these changes were different when compared with the cells containing PtAu NRs irradiated by the 808 nm laser.

**Fig. 7:**
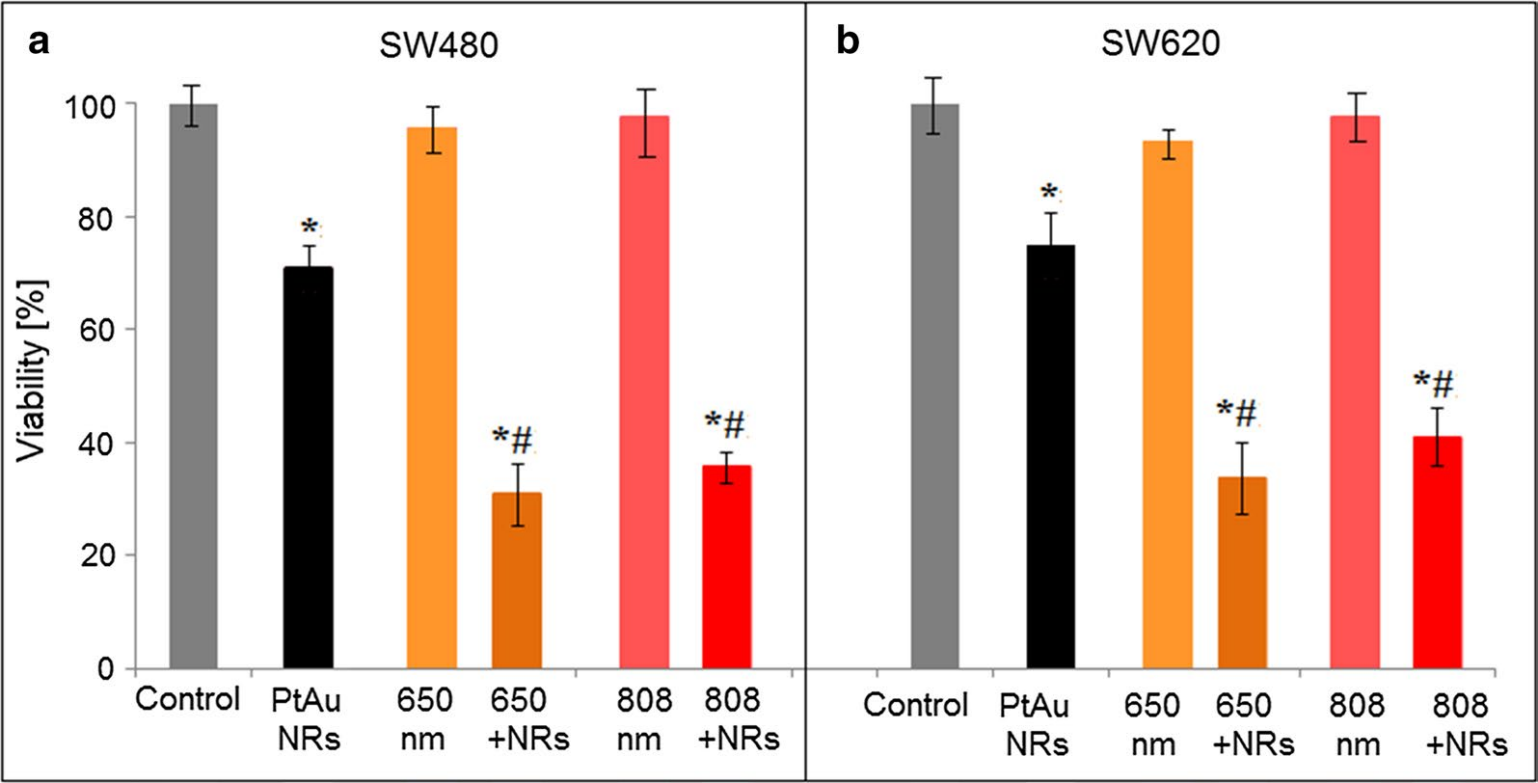
Viability of colon cancer cells: SW480 and SW620 after addition of PtAu NRs and after laser irradiation with and without PtAu NRs. Data was considered as significant when *p < 0.05 vs. control; ^#^p < 0.05 vs. PtAu NRs. Grey color—control samples; black color—cells cultured with PtAu NRs; bright orange—cells irradiated by the 650 nm laser during 5 min; dark orange—cells cultured with PtAu NRs and irradiated by the 650 nm laser during 5 min; bright red—cells irradiated by the 808 nm laser during 5 min; dark red—cells cultured with PtAu NRs and irradiated by the 808 nm laser during 5 min

The MTS assay was used to determine the influence of nanoparticles addition to cells and laser irradiation with and without the presence of PtAu NRs on the viability of cancer cells, Fig. 7.

Figure 7 shows the results of the MTS assay presented by mean ± SEM percentage values of cells viability. Two combinations allowing the comparison of all samples was performed in: *p < 0.05 corresponding to the comparison with the control, and ^#^p < 0.05 corresponding to the comparison with the cells cultured with PtAu NRs. In Fig. 7, statistically significant differences between the investigated samples and the control groups were observed. Addition of the PtAu NRs to the cell cultures caused a 27% and 22% mortality of the SW480 and the SW620 cells, respectively. Furthermore, not statistically significant changes in the viability of cells were observed for samples irradiated by the 650 nm and 808 nm lasers. However, adding PtAu NRs to cells cultures and subjecting these cells to laser irradiation caused an increase of mortality of the cancer cells. The 650 nm laser caused around 65% mortality of both cell lines, while the 808 nm laser caused ~ 60% mortality of cells with PtAu NRs. However, a higher percentage of dead cells in the SW480 line was noticed. When we compare the mortality of cells cultured with PtAu NRs and irradiated by lasers, a ~ 40% higher mortality in both cells lines, were observed for the 650 nm laser irradiated samples and ~ 35% higher mortality when the 808 nm laser was used.

### Photothermal conversion efficiency determined from the time constant

**Fig. 8:**
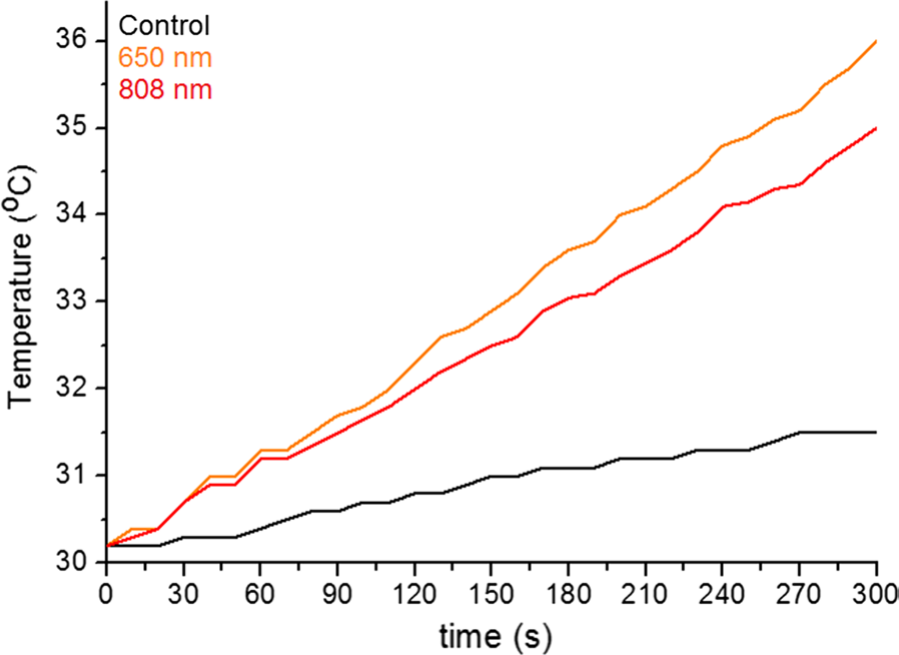
Temperature changes in PtAu NRs solutions during 5 min irradiation by the 650 nm (red line) and the 808 nm (orange line) lasers. The black line corresponds to the water (control) solution

The changes of temperature in the solution with PtAu NRs irradiated by 650 nm and 808 nm lasers, were investigated to determine the efficiency of the photothermal conversion (Fig. 8).

A temperature increase of 5.8 °C in the PtAu NRs solution was observed for the 650 nm laser (Fig. 8, orange line), which is higher than for the 808 nm laser (4.8 °C) (Fig. 8, red line). The smallest increase of temperature was noticed for the control solution: only 1.3 °C.

Moreover, the calculated photothermal conversion efficiency showed that the value of η is higher for the 650 nm laser than for the 808 nm laser, being 62% and 51%, respectively. Furthermore, the highest value (72%) of photothermal efficiency of nanoparticles was obtained for the 650 nm laser. For the laser with the 808 nm wavelength η_NRs_ is 12% lower, than for the 650 nm laser.

## Discussion

High extinction coefficients of Au NPs and Pt NPs in the NIR region make the core/shell nanoraspberries attractive candidates to be used in photothermal therapy [13]. However, the cytotoxicity of platinum itself is quite high, so its surface has to be modified or combined with substances, which are biocompatible [42]. Fortunately, gold has not only excellent photothermal properties, it is also a metal exhibiting a low cytotoxic effect [43, 44]. Herein, a combination of Pt NPs and Au NPs is created by synthesizing Au NPs on Pt NPs forming PtAu NRs, to show their light-absorbing properties in the biological near-infrared window.

STEM HAADF images (Fig. 1a) with the EDX maps (Fig. 1b) showed that larger Pt NPs are surrounded by a dozen of Au NPs. Due to this fact the interaction of the more cytotoxic Pt NPs with cells is hindered. Moreover, the SAED and XRD patterns (Fig. 2) showed that the PtAu NRs have a crystalline fcc structure. The cytotoxic effect of the nanoparticles depends on their crystallinity, because the photocatalytic activity of NPs is directly proportional to the availability of active sites and not to the total surface area of the NPs [45]. For amorphous NPs, a higher cytotoxic effect was observed, therefore for biological applications it is very important that the nanoparticles are crystalline like it is the case of the investigated PtAu NRs [46]. However, after irradiation, a high mortality of cancer cells cultured with PtAu NRs, was observed, in contrast to irradiated cells, cultured however without the nanoraspberries (Fig. 7). These results were also confirmed by the microscopy images of cells with PtAu NRs subjected to irradiation (Fig. 3), showing cells peeled off from the support, indicating their death. It was caused by the increase of temperature in the cancer cells, which was induced by the laser irradiation of the PtAu NRs present in the cell culture. Cancer cells are more sensitive to high temperature than healthy cells. High temperatures during laser irradiation, which are generated by the presence of PtAu NRs (Fig. 8) in cancer cells, lead to necrosis or apoptosis of the latter ones, while in normal cells these high temperatures did not cause significant changes [47–49]. Moreover, the PtAu NRs have high values of photothermal conversion efficiency in the electromagnetic range corresponding to biological near-infrared windows. Shang et al. showed a 70% mortality of cancer cells after 150 s irradiation by an 808 nm laser with addition of Au–Pt NPs [50]. However, they used a laser with ten times higher power, than the one in the present work. Moreover, the Au–Pt NPs synthesized by Shang et al. were composed of larger Au NPs, which were surrounded by more cytotoxic Pt NPs. Furthermore, smaller Pt NPs exhibit a higher active surface, which plays a crucial role in anticancer properties [51].

FTIR (Fig. 4) and FT-Raman (Fig. 6) spectra of cells cultured with PtAu NRs showed that laser irradiation caused chemical changes in the structure of DNA, RNA and phospholipids, which could mean necrosis of cancer cells. Necrosis is characterized by loss of plasma membrane integrity, and the cell membrane consists mainly of phospholipids [52]. Moreover, the laser irradiation effect can trigger detrimental inflammatory and immunogenic responses, inducing necrosis or other kind of cell death [53]. Furthermore, the spectra showed chemical changes in the protein and lipid structures of cancer cells. The high temperature observed due to laser irradiation in the presence of PtAu NRs, caused the protein denaturation [7]. As a result of this process, changes in the structure and function of these chemical compounds, were induced. In FTIR (Fig. 4) and FT-Raman (Fig. 6) spectra of cells cultured with PtAu NRs, also changes in the protein structure were visible. These can be caused by platinum, which has enzymatic properties and can change the functionality of chemical structures building the cells [54].

Summarizing, the synthesized PtAu NRs have a crystalline structure, which caused a decrease of the cytotoxic effect on cancer cells. High values of nanoparticles photothermal efficiency, 72% for the 650 nm laser and 60% for the laser with 808 nm wavelength, suggest that these nanoparticles could be applied as effective light-absorbers in the PTT anticancer therapy.

## Supplementary information

**Additional file 1: Figure S1.** Viability of colon cancer cells: SW480 (a) and SW620 (b) after irradiation by 650 nm and 808 nm lasers during 5, 10 and 15 min. Data was considered as significant when *p < 0.05 vs. Control samples.

## Data Availability

All data generated or analyzed during this study are included in this published article and its additional information files.
