# Supplementary material for: Platinum–gold nanoraspberries as effective photosensitizer in anticancer photothermal therapy
Source: J Nanobiotechnology. 2019 Oct 15;17:107. doi: 10.1186/s12951-019-0539-2 (PMC6794780; doi:10.1186/s12951-019-0539-2)
Supplement: Supplementary file 1 — Additional file 1: Figure S1. Viability of colon cancer cells: SW480 (a) and SW620 (b) after irradiation by 650 nm and 808 nm lasers during 5, 10 and 15 min. Data was considered as significant when *p < 0.05 vs. Control samples. [file 12951_2019_539_MOESM1_ESM.docx]

**Additional file**

**
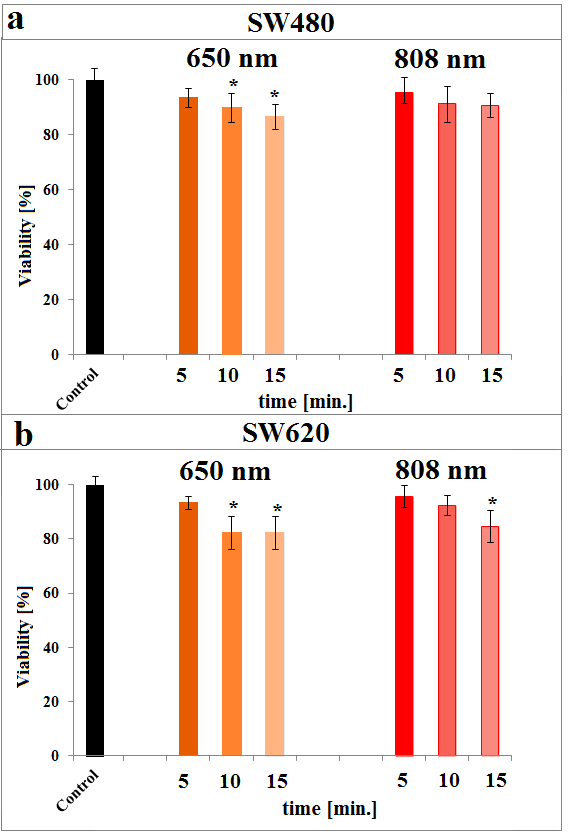
**

Figure S1. Viability of colon cancer cells: SW480 (a) and SW620 (b) after irradiation by 650 nm and 808 nm lasers during 5, 10 and 15 minutes. Data was considered as significant when *p < 0.05 vs. Control samples.

MTS assay showed, that significant changes in the viability of cells are visible in the SW480 and SW620 cells irradiated for 10 and 15 minutes by laser with a 650 nm wavelength. For the 808 nm laser, significant changes in the viability of SW620 cells were visible after 15 minutes irradiation. Therefore, for our experiment, we took 5 minutes irradiation, because we needed to have the same irradiation time for both wavelengths to investigate the influence of PtAu NRs on the mortality of irradiated cells.
